# Inflammation-Related Changes in Mood Disorders and the Immunomodulatory Role of Lithium

**DOI:** 10.3390/ijms22041532

**Published:** 2021-02-03

**Authors:** Kosma Sakrajda, Aleksandra Szczepankiewicz

**Affiliations:** Molecular and Cell Biology Unit, Poznan University of Medical Sciences, 61-701 Poznań, Poland

**Keywords:** mood disorders, inflammation, biomarkers, lithium treatment

## Abstract

Mood disorders are chronic, recurrent diseases characterized by changes in mood and emotions. The most common are major depressive disorder (MDD) and bipolar disorder (BD). Molecular biology studies have indicated an involvement of the immune system in the pathogenesis of mood disorders, and showed their correlation with altered levels of inflammatory markers and energy metabolism. Previous reports, including meta-analyses, also suggested the role of microglia activation in the M1 polarized macrophages, reflecting the pro-inflammatory phenotype. Lithium is an effective mood stabilizer used to treat both manic and depressive episodes in bipolar disorder, and as an augmentation of the antidepressant treatment of depression with a multidimensional mode of action. This review aims to summarize the molecular studies regarding inflammation, microglia activation and energy metabolism changes in mood disorders. We also aimed to outline the impact of lithium on these changes and discuss its immunomodulatory effect in mood disorders.

## 1. Introduction

Mood disorders (also known as affective disorders) are chronic and recurrent psychiatric conditions affecting emotions, motivation, and energy. The alterations of affect caused by mood disorders impair the ability to cope with daily life, including performance at work or school. The wide spectrum of mood disorders can be classified by the severity and range of mood change. The most common include major depressive disorder and bipolar disorder [1,2].

Major depressive disorder (MDD) is characterized by depressed mood, anhedonia, and decreased energy, as well as problems with concentration, feelings of guilt, and in its severe episodes, suicidal thoughts and attempts. MDD affects about 163 million people worldwide [3], and the World Health Organization (WHO) ranked MDD as the single largest contributor to global disability [4].

Bipolar disorder (BD), previously known as manic depressive illness, is a chronic, multifactorial and biphasic condition characterized by episodes of elevated mood and increased motor efficiency—episodes of mania or hypomania—and episodes of depression expressed as low mood and decreased energy [5,6]. BD affects approximately 45 million people worldwide [3]. Due to an increased risk of suicide, and other comorbidities such as cardio- and cerebrovascular diseases, endocrine, and metabolic disorders, bipolar disorder shortens life expectancy by 11 years in females and 10 years in males [7,8].

## 2. Pathophysiology of Mood Disorders

Molecular biology studies have enabled rapid progress in elucidating the pathophysiology of mood disorders. Several theories were formulated, including an imbalance in monoaminergic neurotransmission, stress response, disturbances in the hypothalamic–pituitary–adrenal (HPA) axis, and neurotrophic pathways alterations. Changes were also observed within the immune system, not only in the central nervous system, but also in the periphery, including the production of inflammatory cytokines that lead to microglia activation. Such activation may also be induced as a result of alterations in mitochondrial activity and energy metabolism within the central nervous system.

### 2.1. Inflammation Theory

Inflammation is a response of the organism to harmful stimuli, and conditions such as physical injury or infection. The immune system triggers inflammation to maintain or restore the disturbed homeostasis. Inflammatory responses are mediated by cytokines, and this group includes interleukins (IL), chemokines, interferons (IFN), tumor necrosis factors (TNF), and lymphokines. Cytokines are produced by immune cells such as macrophages, T cells, B cells or mast cells, and non-immune cells such as endothelial cells, fibroblasts, and various stromal cells. The cytokines act through receptors modulating the immune responses by a self- (autocrine), locally (paracrine), or distally (endocrine) determined manner, either enhancing immune response or showing an anti-inflammatory effect by affecting various biological processes [9].

Dysfunction of the immune system and chronic inflammation have been implicated in the pathophysiology of numerous psychiatric disorders [10,11]. In 1990, Maes et al. [12] revealed a correlation between the cytokines level in blood and major depressive disorder. At the same time, Smith et al. [13] described the relationship between depression and pro-inflammatory cytokines produced by macrophages, which was the basis of the inflammatory theory that assumes the crucial role of immune cells present in the central nervous system (CNS)—resident macrophages called microglia [14]. The physiological microglia’s role is the active scanning of CNS and responding to tissue injury and infections. The microglia hypothesis of mood disorders describes the microglia’s over-activation in the brain regions controlling mood and cognition, e.g., amygdala, hippocampus and prefrontal cortex [15,16]. In bipolar disorder, immune disturbances underlie the disrupted white matter microstructure and functional connectivity. Higher inflammatory cytokines, higher macrophage/monocyte inflammatory activation patterns and reduced T cell activity correlate with white matter disruption [17,18,19]. High peripheral levels of pro-inflammatory cytokines, reflecting M1 macrophages, also correlate with poor antidepressant response [18]. Interestingly, a study by Benros et al. revealed that autoimmune and infectious diseases increases the risk of future mood disorder diagnosis [20]. Therefore, research on the role of microglia activation may be a promising direction to take in the studies on mood disorders pathogenesis.

### 2.2. Cytokines as Markers of Microglial Activation

**Figure 1:**
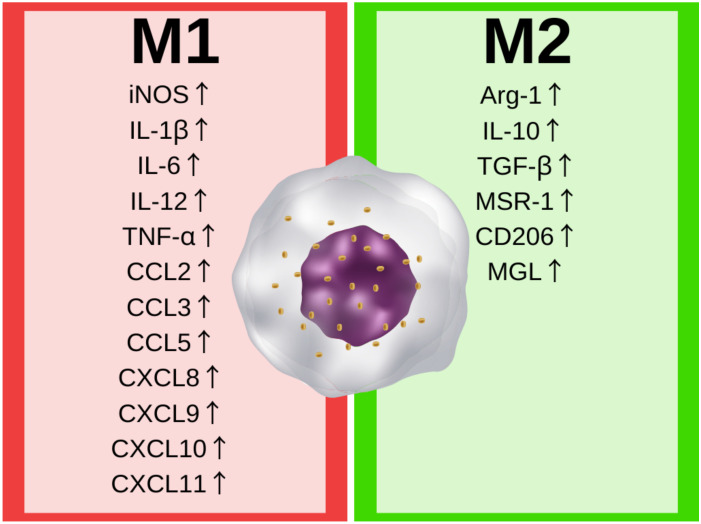
Characteristic molecular markers elevated in M1 and M2 phenotype. iNOS—inducible nitric oxide synthase; IL-1β—interleukin 1 beta; IL-6—interleukin 6; IL-12—interleukin 12; TNF-α—tumor necrosis factor alpha; CCL2—C-C motif chemokine ligand 2 or monocyte chemoattractant protein1; CCL3—C-C motif chemokine ligand 3 or macrophage inflammatory protein 1-alpha; CCL5—C-C motif chemokine ligand 5; CXCL8—C-X-C motif chemokine ligand 8 or interleukin 8; CXCL9—C-X-C motif chemokine ligand 9; CXCL10—C-X-C motif chemokine ligand 10; CXCL11–C-X-C motif chemokine ligand 11; Arg-1—arginase-1; IL-10—interleukin 10; TGF-β—transforming growth factor beta; MSR-1—macrophage scavenger receptor 1; CD206—mannose receptor (or cluster of differentiation 206); MGL—galactose-type lectin.

Macrophages and their counterparts are involved in the innate immune response in the central nervous system. The microglia—resident macrophages of the CNS—account for about 5–10% of the brain cells, and are the most common immune cells in the CNS [21]. The central physiological role of microglia is to maintain homeostasis by the induction and resolution of inflammation in response to exogenous (viral or bacterial) or endogenous (DNA, RNA, protein) signals by secreting several soluble factors, such as cytokines, chemokines, and neurotrophic factors, necessary for the CNS immune response and tissue repair processes [22,23]. The stimuli are recognized by pattern-recognition receptors (PRRs), the protein receptors on the microglia. The recognition of endogenous danger molecules—damage-associated molecular patterns (DAMPs)—or exogenous molecules frequently found in pathogens—pathogen-associated molecular patterns (PAMPs)—activates the microglia cells’ mechanism of adaption to stimuli, resulting in changes in their phenotype. The microglia phenotype can be generally assigned to the well-known M1/M2 macrophages paradigm. Classically activated (M1) macrophages are characterized by the secretion of pro-inflammatory cytokines and their response to the presence of a potential pathogen, while on the other hand, alternatively activated (M2) macrophages are involved in tissue repair and regeneration [22,24]. The two main phenotypes of macrophages are generally characterized by pathways regulating the metabolism of arginine. M1 macrophages upregulate inducible nitric oxide synthase (iNOS) expression, leading to L-arginine’s conversion to nitric oxide (NO) that can promote tissue injury at cytotoxic levels. Alternatively activated (or M2) macrophages upregulate arginase-1 (Arg-1), and metabolize L-arginine to L-citrulline, which results in the production of prolines and polyamines promoting tissue repair, proliferation, and the synthesis of extracellular matrix [22]. However, changes in arginine metabolism lead to important expression and secretion differences between phenotypes. The main differences in the secreted immune factors and expressed genes are shown in Figure 1.

The classical activation of macrophages is induced by pathogens (by peptidoglycans, lipopolysaccharide, or flagellin on the surface of pathogens). The recognition of pathogens, or pathogen-like stimuli, induces the expression of pro-inflammatory interleukins, including, amongst others, IL-1β, IL-6, IL-12, TNF-α, CCL2 (or Monocyte Chemoattractant Protein 1 [MCP-1]), CCL3 (or Macrophage Inflammatory Protein 1-α [MIP-1-α]), CCL5, CXCL8 (or IL-8), CXCL9 CXCL10 and CXCL11 [22,25]. These cytokines lead to the further activation of the immune system, e.g., interleukin 12 stimulates T helper 1 (Th1) cell development and the secretion of IFN-γ that perpetuates the phenotype of M1 macrophages [24].

In contrast, the alternative activation of macrophages in the M2 phenotypes is caused by IL-4 produced by T helper 2 (Th2), prostaglandins, apoptotic cells, or IL-10, which upregulate Arg-1 expression and activity. Moreover, the M2 macrophages are characterized by the production of anti-inflammatory cytokines, e.g., IL-10 and transforming growth factor-beta (TGF-β). The M2 phenotype leads to a reduction in inflammation, and promotes tissue regeneration and elevated expressions of scavenger (MSR-1), mannose (CD206), and galactose-type (MGL) receptors. [24,26]. However, the M2 phenotype is not homogenous in the human body, and new conceptions defining the phenotype via several subtypes, depending on cell environment and activity, are receiving increasing attention [27].

Recent findings in the molecular biology and immunology of mood disorders have supported an association between the inflammatory responses of the immune system and macrophages’ phenotype switch upon microglial activation, as well as an increased risk of mood disorders. Studies showed altered levels of cytokines and chemokines associated with macrophage polarization in both MDD and BD measured in peripheral blood or the central nervous system (cerebrospinal fluid). The results of the studies analyzing the pro- and anti-inflammatory cytokines levels in mood disorders (major depressive disorder and bipolar disorder), either during manic or depressive episodes or in a euthymic state, are summarized in Table 1.

Despite a large number of studies and meta-analyses comparing the levels of inflammatory markers during the course of mood disorders, the data are still inconsistent. The reason for heterogeneity in these results may be the common use of a cross-sectional model instead of a prospective study, revealing the changes in the patient’s inflammatory markers’ shift, as well as the heterogeneity between the studies’ designs (e.g., different sample sizes, populations and ethnic groups, diagnostic criteria, disease duration or provided pharmacotherapy). Interestingly, some studies revealed the upregulation of both pro- and anti-inflammatory markers in differently polarized macrophages. Further comparison of the peripheral and central levels of pro- and anti-inflammatory cytokines in the context of the disease state, as well as normotymic drugs and comorbid disorders, seems essential to better understand the inflammation shifts in the course of the mood disorders.

The inconsistencies were also observed in brain samples and were recently summarized by a systematic review by Giridharan et al. [54]. They analyzed the data from brain inflammation studies evaluating microglia, astrocytes, cytokines, chemokines, adhesion molecules, and other inflammatory markers in postmortem BD brain samples. They reported that out of 51 studies in brain tissue, most of them confirmed neuroinflammation in BD postmortem samples. However, a large number of these studies did not evaluate the presence of infiltrating peripheral immune cells in the CNS parenchyma, cytokines levels, and microglia activation in the same postmortem brain sample. Moreover, the heterogeneity in postmortem samples regarding postmortem interval, studied brain area, age at diagnosis, the treatment, cause of death and comorbidities might underlie the observed inconsistencies. Therefore, more prospective studies, including precisely defined phenotypes, are necessary to elucidate how changing inflammatory marker levels influence the course of mood disorders. More in-depth research on the activated microglia phenotype would also be valuable in postmortem brain samples analyzed by cell type and brain region, to confirm the presence of neuroinflammation in the pathogenesis of mood disorders.

### 2.3. Energy Metabolism Markers of Microglia Activation

Mitochondria are intracellular organelles that regulate energy metabolism, the production of reactive oxygen species (ROS), cellular signaling, Ca^2+^ homeostasis, and apoptosis. Studies revealed that the mitochondrial dysfunction and damage play a crucial role in the development of neurodegenerative diseases, including amyotrophic lateral sclerosis, Alzheimer’s disease, Parkinson’s disease, Huntington’s disease or multiple sclerosis, by enhancing innate and adaptive immune responses that result in neuroinflammation [55]. The dysfunction of mitochondria and impaired energy metabolism were also reported in the pathophysiology of mood disorders such as bipolar disorder and major depressive disorder [56,57,58,59]. The mitochondrial hypothesis suggests that changes in the cell energy metabolism activate inflammasome, increase cytokines production and further lead to apoptotic cell death. The changes underlying mitochondrial dysfunction in bipolar disorder include decreased ATP synthesis, disrupted electron transport chain (ETC), a switch from oxidative phosphorylation (OXPHOS) to aerobic glycolysis, increased lactate levels and oxidative stress markers, and reduced antioxidant capacity (decreased glutathione peroxidase and superoxide dismutase) [60,61,62,63,64]. Mitochondrial dysfunction and increased lactate levels were also observed recently in an adolescent population of BD patients, suggesting that the impediment of mitochondrial oxidative phosphorylation and a shift towards anaerobic respiration and lactate production occur at the early stage of disease development [65]. These alterations further support the role of mitochondrial dysfunction leading to a shift towards anaerobic metabolism, and an associated elevated risk for cellular injury. These studies also suggested that, taking into account the similarities between mitochondria and bacteria, it is possible that the mitochondrial contents released to the cytoplasm of microglial cells are recognized as pathogen-associated molecular patterns (PAMPs) or damage-associated molecular patterns (DAMPs), which further activate pattern recognition receptor (PRR) signaling, thus enhancing inflammation [66].

The underlying cause of the disturbed energy metabolism and altered mitochondrial activity may be the microglia activation itself, which results in polarization towards either the M1 or the M2 phenotype. The M2 microglia (considered non-activated microglia) are characterized by low glucose consumption and oxidative phosphorylation for ATP synthesis. The pro-inflammatory M1 phenotype contributes to the switch from oxidative to glycolytic metabolism, with high glucose uptake leading to lactate, NO and citrulline production with activation of the penta phosphate pathway (PPP) [67,68]. These changes in energy metabolism increase pro-inflammatory interleukins levels and enhance the production of ROS that are harmful to all cellular components, further progressing the inflammation and energy metabolic changes.

Translocator protein (TSPO) is one of the mitochondrial neuroimmune markers of microglia activation. TSPO ligands are used in positron emission tomography (PET) imaging as a biomarker of microglia activation in neurodegenerative diseases [69,70,71,72]. Taking into account the neuroinflammatory theory of mood disorders that assumes that the activation of microglia contributes to disease phenotype, we suggest that this protein may also be an important marker of neuroinflammation in mood disorders. TSPO is an 18 kDa protein localized on the outer mitochondrial membrane. It is responsible for mitochondrial homeostasis by direct interaction with the voltage-dependent anion channel (VDAC) and the adenine nucleotide transporter (ANT), thus contributing to energy production, Ca^2+^ signaling, ROS generation and apoptosis [73,74]. Recent studies have described the effect of an elevated expression of TSPO and VDAC on both gene and protein levels in the group of BD and MDD patients, as compared to controls [40,75,76]. Scaini et al. indicated that the upregulation of TSPO-related proteins (TSPO and VDAC) is one of the causes of the inflammasome activation of the NLR family pyrin domain containing 3 (NLRP3), by increasing ROS production and Ca2+ dysregulation in bipolar disorder patients [75]. Inflammasome complexes are multi-domain, cytoplasmatic protein complexes composed of three components: a cytosolic pattern recognition receptors (PRR) (detecting molecules typical for the pathogenic activity), caspase-1, and an adaptor protein (responsible for caspase activation) [77]. Inflammasome complexes are activated by a wide range of stimuli, including, e.g., bacterial infections, extracellular ATP, but also mitochondrial content. This activation stimulates NF-κB signaling, which induces NLRP3 inflammasome complex assembly and activates caspase 1 [77]. Activated caspase 1 promotes the cleavage of pro-interleukins into mature interleukins (IL-1β and IL-18) [77,78], and their secretion, leading to chronic inflammation [75,79,80]. A study by Alcocer-Gómez et al. described the increased level of mitochondrial ROS and the increased gene expression of NLRP3 and caspase-1 in the MDD patients not treated with antidepressants [81]. Further in vivo studies by Pan et al. described the upregulation of NLRP3, NF-κB, caspase-1 and IL-1β gene expression after inducing depressive-like behavior via a chronic unpredictable mild stress protocol. These alterations in gene expression levels were restored after the administration of antidepressant treatment [82]. Further studies by Wong et al. showed that the inhibition of inflammasome signaling by caspase-1 knock-out reduced depression-like behavior in response to chronic restraint stress in animals, supporting the important role of NLRP3 inflammasome in the pathogenesis of depressive-like behavior [83].

Although changes in energy metabolism in microglia seem to be an important factor in the development of neuroinflammation, more studies are necessary to establish if these metabolic shifts are the cause or the result of the inflammation.

## 3. Lithium Influences Inflammation and Energy Metabolism

Lithium is the smallest monovalent cation used in the pharmacotherapy of psychiatric disorders. For over 70 years, lithium has been an efficient mood stabilizer with proven anti-manic and antidepressant properties [84]. Despite proven long-term efficacy in treating acute episodes of mania and depression, as well as prophylactic properties, the exact molecular mechanism of action is not fully understood. Despite the well-known modes of lithium action, including GSK-3β inhibition and the phosphatidylinositol pathway, previous studies have also indicated its immunomodulatory potential, suggesting that lithium may decrease the levels of pro-inflammatory cytokines in mood disorders, either in the periphery or in the central nervous system, and that these changes are related to its therapeutic efficacy [85,86,87].

The best-known mode of action for lithium is the inhibition of glycogen synthase kinase-3β (GSK-3β), ubiquitously expressed in the tissues and associated with inflammation. The GSK-3β can affect a variety of transcription factors, including NF-κB, which is crucial for innate immune response and regulates cytokines production [88]. The decreased transcriptional activity of NF-κB by GSK-3β inhibition—including lithium treatment—reduces the production of pro-inflammatory mediators such as IL-1β, IFN-γ, IL-6, and MCP-1, associated with M1 macrophages, and increases the production of anti-inflammatory cytokines [88,89,90]. Another potential downstream target of GSK-3β is a signal transducer and activator of transcription (STAT). A previous study showed that the inhibition of GSK-3β by lithium resulted in the reduced activation of STAT and the reduced secretion of pro-inflammatory cytokines [91,92]. Martin et al. [89] showed that the inhibition of GSK-3β upregulated anti-inflammatory IL-10 production and reduced pro-inflammatory cytokines, such as IL-1β, IL-6, TNF, IL-12, and IFNγ, associated with M1 macrophages by affecting Toll-like receptor-mediated production. The clinical study by Rapaport et al. reported decreased levels of inflammatory markers in serum in rapid cycling bipolar patients after 30 days of lithium treatment [86]. Boufidou et al. presented a study comparing the cytokine levels (IL-2, IL-6, IL-10 and IFN-gamma) in the peripheral blood of healthy volunteers and lithium-naive BD patients before and after lithium treatment [87]. Although they did not observe differences in the cytokine levels at the beginning of lithium treatment, after three months on lithium, the patients presented a significant decrease in all analyzed cytokines compared to controls. A study analyzing ex vivo interleukin IL-1β and IL-6 secretion in BD patient-derived monocytes stimulated with LPS showed that lithium treatment decreased the production of IL-6 compared to the non-treated control [93]. Recently, Wu et al. [94] analyzed immunophenotypes of BD patients and found that they had significantly higher percentages of total T cells, CD4^+^ T cells, activated B cells, and monocytes than healthy controls, whereas the treatment of patient-derived PBMCs with lithium in vitro increased the percentage of CD14^+^ monocytes and dendritic cells. The authors suggested that lithium plays an immunomodulatory role in CD14^+^ monocytes and dendritic cells [94]. A study from our center showed that long-term lithium decreased the expression of glial and pluripotency markers (in particular Oct-4, Sox-2, GFAP and Olig1) in peripheral blood, suggesting that lithium can reduce ongoing inflammatory processes in bipolar disorder [95].

The observations from clinical studies were also confirmed in vitro. The study by Nahman et al. [96] showed that pre-treatment of the rat primary glial cells with lithium suppressed LPS-induced inflammation, including reduced levels of M1 macrophages markers such as TNF-α, IL-1β and iNOS expression. Although the results indicated the anti-inflammatory potential of lithium, they were obtained by using concentrations higher than the therapeutic dose [96]. A study by Yuskaitis et al. [90] analyzed the influence of lithium and other GSK-3β inhibitors on microglia activation and migration, and they showed that lithium decreased microglial migration in mouse BV-2 cells as well as attenuating the injury-induced migration of microglia in situ in hippocampal mouse slices. Lithium also decreased inflammatory markers IL-6, NO and iNOS in microglial cells stimulated with LPS, showing the anti-inflammatory potential of lithium [90]. More recently, Dong et al. [97] confirmed that lithium treatment influenced microglial activation. The pretreatment of primary microglial cells with lithium followed by LPS stimulation inhibited LPS-induced microglial activation and pro-inflammatory cytokine production. This suppression resulted from LPS-induced toll-like receptor 4 (TLR4) downregulation and proceeded via inhibition of the PI3K/Akt/FoxO1 signaling pathway [97].

The anti-inflammatory effect of lithium was also confirmed in the recent animal study by Adams et al. [98]. They observed that chronic lithium treatment (12 weeks) decreased pro-inflammatory cytokines’ levels (IL-1β, IL-6 and RANTES) in plasma and the orbitofrontal cortex, leading to reduced motor impulsivity in rats. The authors suggested that improved impulse control deficits in patients treated with lithium may result from the reduced influence of pro-inflammatory signaling on neuronal activity.

The anti-inflammatory potential of lithium was also considered in a recent study of the olanzapine (OLZ) and lithium co-treatment. Olanzapine is a second-generation antipsychotic drug with side effects, including metabolic alteration associated with low-grade systemic inflammation. The in vitro study by Fernandes et al. [99] used the activated RAW 267.7 macrophages treated with OLZ or OLZ and lithium, and evaluated the oxidation and inflammation at the gene and protein expression level. The macrophages treated only with OLZ showed increased proliferation and higher levels of oxidative markers and pro-inflammatory cytokines, with reduced anti-inflammatory IL-10 level. Co-treatment with lithium resulted in a significantly reduced level of oxidative and inflammatory markers, and an increased IL-10 level.

Despite studies describing the anti-inflammatory role of lithium, some pieces of evidence have showed also its pro-inflammatory action. Petersein et al. analyzed the blood collected from healthy subjects stimulated ex vivo with phytohemagglutinin (PHA), murine anti-human CD3 monoclonal antibody OKT3 and 5C3 monoclonal antibody (OKT3/5C3), as well as non-stimulated blood, followed by incubation with different psychotropic drugs combinations: lithium without antidepressant, co-treatment with lithium and antidepressant, antidepressant without lithium and no drug as a negative control. They showed that lithium alone or in combination with antidepressants increased several pro-inflammatory cytokines, including IL-1β, IL-6 and TNF-α, independently of stimulation [100]. However, it is not known if the same pro-inflammatory action would also be observed in the blood collected from mood disorder patients.

In regard to energy metabolism and mitochondrial dysfunction, the early in vitro study in cultured cerebellar granule neurons showed that lithium increased the mitochondrial calcium concentrations that desensitized mitochondria to depolarization [101]. Lithium was also described as a modulator of ETC and OXPHOS pathway activity, and enhanced the oxidative phosphorylation in human brain tissue [102]. A previous clinical study reported increased complex I activity and enhanced oxidative phosphorylation after lithium treatment, and this increase positively correlated with plasma lithium levels in BD patients [103].

On the other hand, the recent in vitro study analyzing the effects of different mood stabilizers on pig brain mitochondria showed no inhibitory effects of lithium on complex I- and II-linked respiration [104]. However, in cultured human neurons (SH-SY5Y), chronic lithium treatment (25–50 weeks) increased resistance to oxidative stress, and enhanced glucose consumption and glycolysis activity [105]. These results suggested the neuroprotective effect of chronic lithium exposure via the improved resistance of neurons to oxidative stress. A recent in vitro study performed in two rodent cell lines (murine microglial N9 and rat pheochromocytoma PC12) showed that lithium prevented ATP-induced cell death in PC12 neuronal cells, but in the N9 microglial cell line lithium treatment did not prevent the ATP-induced microglial switch to the M1 phenotype [106].

Previous preclinical studies showed that lithium reversed hyperactivity and risk-taking behavior after repeated injections of amphetamine [107,108], and thus was considered a validated model of mania in rats [109,110,111]. Therefore, studies using this model of mania confirmed that lithium prevented the amphetamine-induced inhibition of ETC complexes I, II, III, and IV in the hippocampus, striatum and prefrontal cortex. Lithium pre-treatment also attenuated hyperactive behavior, suggesting that restoring mitochondrial functioning improved the behavioral changes induced by amphetamine [112].

Recent clinical studies confirmed changes in OXPHOS and ETC activity. The co-expression network analysis of RNA-seq data from the whole blood of lithium-treated BD patients reported the significant over-representation of genes involved in mitochondrial functioning (specifically ETC and OXPHOS related genes), and the expression of these genes was lower in better lithium responders relative to poor lithium responders, thus indicating that lithium is involved in the regulation of these mitochondrial functioning pathways [113]. A recent study by Machado-Vieira et al. suggested that a shift from aerobic to anaerobic metabolism underlies mitochondrial dysfunction and energy metabolism alterations in bipolar disorder. Using proton magnetic resonance spectroscopy (H-MRS), they measured brain lactate in vivo in the cingulate cortex of BD patients during a depressive episode and after six weeks of lithium therapy to evaluate mitochondrial and metabolic dysfunction. They showed that during the depressive episode, lactate production increased and mitochondrial metabolism changed towards anaerobic glycolysis, whereas lithium therapy restored the lactate level in the brain and improving mitochondrial respiration [114]. Those results confirmed the important role of lithium in mood disorder therapy by suppressing the CNS neuroinflammation, not only as a potential anti-inflammatory agent, but also via its potential impact on the brain energy metabolism. Despite the long history of using lithium as a mood stabilizer, its influence on the immune system and energy metabolism is still not fully understood. Further studies considering its immunomodulatory potential may shed light on its role in the pathogenesis of mood disorders.

## 4. Anti-Manic and Antidepressant Properties of Anti-Inflammatory Drugs

The emerging evidence of neuroinflammation in the pathophysiology of mood disorders has led to several meta-analyses describing the efficacy of anti-inflammatory agents therapy for MDD and BD patients. A recent meta-analysis including 1610 MDD patients from 26 randomized controlled trials suggested the potential use of antidepressants in patients receiving non-steroidal anti-inflammatory drugs (NSAIDs) compared to the placebo group [115]. Moreover, subgroup analysis indicated a significant reduction in the severity of depressive symptoms in groups treated via monotherapy, as well as via adjunctive treatment with anti-inflammatory agents [115]. Another meta-analysis investigating the effect of adjunct treatment with NSAIDs, omega-3 fatty acids, N-acetylcysteine and pioglitazone on bipolar disorder indicated a significant effect on reducing depressive symptoms. However, these anti-inflammatory drugs were not effective in reducing mania symptoms after adjunctive anti-inflammatory therapy [116]. Another meta-analysis by Husain et al. [117] showed the beneficial effect of anti-inflammatory medications—with NSAIDs, N-acetyl cysteine, cytokine inhibitors or minocycline—on manic and depressive symptoms measured as post-treatment symptoms’ severity; however, the authors emphasized the small number of studies investigating the effect of the anti-inflammatory treatment of mania [117].

Based on the results of the presented meta-analyses, further studies analyzing the effects of anti-inflammatory agents on the course of mood disorders, particularly on the severity of manic episodes, seem necessary in order to better understand the role of inflammation in mood disorders.

## 5. Conclusions

Alterations of the pro- and anti-inflammatory markers underlie the changes in the immune system observed in mood disorders. Increased levels of M1-associated pro-inflammatory cytokines and chemokines indicate the potential role of microglia polarization as a pathological factor of mood disorders. The energy metabolic changes in mood disorders are another source of immune system alterations, leading to the state of neuroinflammation and microglial cells’ pro-inflammatory polarization. However, few studies so far have deeply investigated the energy metabolism-related pathway and its impact on microglia activation in the course of mood disorders. Lithium, a well-described and effective mood stabilizer, showed an immunomodulatory effect in previous studies. However, there is a lack of studies correlating the cytokines and metabolic-related genes levels with lithium treatment in the clinical setting. Therefore, more prospective studies evaluating the effects of lithium treatment, and the levels of neuroinflammatory and energy metabolism markers described above, may shed light on the molecular etiology and the course of mood disorders, as well as lithium’s mode of action.

## Figures and Tables

**Table 1:** Changes in pro- and anti-inflammatory interleukins in mood disorders.

Interleukin	Major Depressive Disorder	Bipolar Disorder		
		**Depression**	**Euthymia**	**Mania**
Interleukin 1 beta	↑ IL-1β [28]	↑ IL-1β [29]	↑ IL-1β [30,31,32,33]	↓ IL-1β [29,34]
	↔ IL-1β [35,36,37,38]	↔IL-1β [34]	↔ IL-1β [39]	
Interleukin 6	↑ IL-6 [32,35,36,37,40]	↑IL-6 [29,34]	↑ IL-6 [32,33,41]	↑IL-6 [29,32,41,42,43,44]
			↔IL-6 [38,39]	↓IL-6 [34]
Soluble form of interleukin 6 receptor	↑sIL-6R [45]	N/A	↑sIL-6R [30]	N/A
Tumor necrosis factor alpha	↑TNF-α [35,36,37,40]	↑TNF-α [29,34,42]	↑TNF-α [30,33]	↑TNF-α [29,30,41,42,43,44,46]
			↔TNF-α [31,38]	
C-C motif chemokine ligand 2	↑CCL2 [37,47]	↑CCL2 [29]	↑CCL2 [45,48,49]	↑CCL2 [29]
		↔CCL2 [30]	↔CCL2 [30,38]	↔CCL2 [30]
			↓CCL2 [50]	
C-C motif chemokine ligand 3	↔CCL3 [37,47]	N/A	↑CCL3 [50]	N/A
			↔CCL3 [51]	
C-C motif chemokine ligand 5	↔CCL5 [37,47]	N/A	N/A	N/A
C-X-C motif chemokine ligand 8	↑CXCL8 [47]	↑CXCL8 [42]	↑CXCL8 [33,42]	↑CXCL8 [42]
			↔ CXCL8 [30]	
			↓CXCL8 [52]	
C-X-C motif chemokine ligand 9	↔ CXCL9 [47]	↓CXCL9 [53]	↑CXCL9 [52]	↓CXCL9 [53]
			↔CXCL9 [53]	
Interferon gamma	↔IFN-γ [35,36]	N/A	↑IFN-γ [33]	↑IFN-γ [43]
	↓IFN-γ [37]		↔IFN-γ [39,41]	
Interleukin 4	↔IL-4 [35,36]	↑IL-4 [29]	↑IL-4 [39,50]	↑IL-4 [29,34]
			↔IL-4 [38,41]	↔IL-4 [41] ↓IL-4 [44]
Transforming growth factor beta	N/A	↑TGF-β [53]	↑TGF-β [33]	↑TGF-β [53]
			↔TGF-β [53]	
			↓TGF-β [50]	

↑—a significant increase in the interleukin level; ↓—a significant decrease in the interleukin level; ↔—no significant changes in the interleukin level. N/A—data not available.

## Data Availability

Not applicable.
